# Fabrication of polylactic acid/paclitaxel nano fibers by electrospinning for cancer therapeutics

**DOI:** 10.1186/s13065-020-00711-4

**Published:** 2020-10-23

**Authors:** H. Y. Chi, Vincent Chan, Chuan Li, J. H. Hsieh, P. H. Lin, Ya-Hui Tsai, Yun Chen

**Affiliations:** 1 Division of Cardiovascular Surgery, Department of Surgery, Taoyuan Armed Forces General Hospital, Taoyuan, 32551 Taiwan; 2 Department of Biomedical Engineering, Khalifa University, PO Box 127788, Abu Dhabi, United Arab Emirates; 3 Department of Biomedical Engineering, National Yang Ming University, Taipei, 11221 Taiwan; 4 Department of Materials Engineering, Ming Chi University of Technology, Taishan, New Taipei City, 24301 Taiwan; 5 Department of Surgery, Far Eastern Memorial Hospital, Banqiao, New Taipei City, 22060 Taiwan

**Keywords:** Polylactic acid, Paclitaxel, Electrospinning, Spin coating, Human colorectal carcinoma

## Abstract

Polylactic acid (PLA) is a thermoplastic and biodegradable polyester, largely derived from renewable resources such as corn starch, cassava starch and sugarcane. However, PLA is only soluble in a narrow range of solvents such as tetrahydrofuran, dioxane, chlorinated solvents and heated benzene. The limited choices of solvent for PLA dissolution have imposed significant challenges in the development of specifically engineered PLA nanofibers with electrospinning techniques. Generally, the electrospun polymeric materials have been rendered with unique properties such as high porosity and complex geometry while maintaining its biodegradability and biocompatibility for emerging biomedical applications. In this study, a new anticancer drug delivery system composed of PLA nanofibers with encapsulated paclitaxel was developed by the electrospinning of the respective nanofibers on top of a spin-coated thin film with the same chemical compositions. Our unique approach is meant for promoting strong bonding between PLA-based nanofibers and their respective films in order to improve the prolonged release properties and composite film stability within a fluctuative physiochemical environment during cell culture. PLA/paclitaxel nanofiber supported on respective polymeric films were probed by scanning electronic microscope, Fourier transform infrared spectrometer and water contact measurement for determining their surface morphologies, fibers’ diameters, molecular vibrational modes, and wettability, respectively. Moreover, PLA/paclitaxel nanofibers supported on respective spin-coated films at different loadings of paclitaxel were evaluated for their abilities in killing human colorectal carcinoma cells (HCT-116). More importantly, MTT assays showed that regardless of the concentrations of paclitaxel, the growth of HCT-116 was effectively inhibited by the prolonged release of paclitaxel from PLA/paclitaxel nanofibers. An effective prolonged delivery system of paclitaxel based on PLA nanofiber-based film has demonstrated exciting potentials for emerging applications as implantable drug delivery patch in post-surgical cancer eradication.

## Introduction

Polylactic acid (PLA) is a condensation thermoplastic elastomers with demonstrated low cytotoxicity and good biodegradability and has similar properties compared to those of polypropylene, polyethylene, or polystyrene. Moreover, PLA is an aliphatic (non-aromatic) polymer with a glass transition temperature of around 60 °C and melting points between 130 and 180 °C. Interestingly, the usage of PLA is highly sustainable because its monomers can be obtained from various types of agricultural by-products such as sugarcane, corn starch or cassava roots and can be later on reused as carbon sources for plants after degradation and decomposition. As a result, PLA is relatively cost-effective for large scale production through direct condensation of lactic acid monomers (~ 100–160 °C) or ring-opening polymerization of lactide on metal catalysts. Most importantly, raw material of PLA can be fabricated into different sizes and shape by various common fabrication techniques such as plastic extrusion, casting, injection molding and spin coating or even 3D printing [1–6]. Thus PLA is an attractive platform for various emerging applications in drug delivery, gene therapy and regenerative medicine.

Paclitaxel (PLX) is an organic compound extracted from the bark of a Pacific yew tree known as *Taxus brevifolia*. It belongs to the taxane family which serves as antineoplastic drugs by inhibiting the disassembly of microtubules in cancerous cells through the binding to intracellular GDP-bound tubulins. The binding as mentioned above directly destabilizes the microtubules by stopping the de-polymerization of β-tubulin dimers from microtubules, leading to the disruptions of cell division during mitotic and interphase cellular functions [7–10]. PLX is a prescribed-only, chemotherapeutic medication to treat a few types of cancer. In clinical setting, PLX currently plays a central role as a chemotherapy medication in the treatment of cervical cancer, breast cancer, ovarian cancer, lung cancer, pancreatic cancer, and Kaposi sarcoma. In general, PLX in its original form or newer albumin-bound formulation is usually administrated to patients by intravenous (IV) injection or infusion. The high efficacy of PLX in anticancer therapy has been proven by the absence of elevated liver enzymes without leading to acute liver injury [11–13]. On the other hand, the current formulation of PLX includes Kolliphor which often causes allergic reactions to patients.

On top of the ideal properties for PLA for biomedical applications, random, PLA nanofiber offers complex 3-dimensional microscopic and nanoscale structures for the encapsulation of drugs and hosting of regenerative cells [6]. Moreover, the recent success in using albumin to bound PLX into sub-micron particle, emerging drug delivery system like PLA nanofiber should offer an promising alternative for the prolonged delivery of PLX. In order to evaluate the performance of PLA nanofiber for prolonged PLX delivery, PLA/PLX nanofibers in the form of membrane were fabricated from PLA/PLX mixed solution by electrospinning on top of PLA/PLX thin film with the same composition by spin coating. PLA/PLX thin film spun coated on glass should not lead to significant interference with drug delivery studies involving the supported PLA/PLX nanofibers lying above. Since both thin film and nanofibers as mentioned above had the same chemical composition, strong bonding between the two forms of PLA/PLX should be naturally enforced upon the nanofiber deposition on thin film. To the best of our knowledge, the fabrication of drug delivery patch of PLA/PLX nanofibers immobilized on the same polymer thin film against the change of PLX concentrations aiming for anti-cancer drug delivery has not been reported previously. In this study, the structure, morphology, and compositions of the PLA/PLX nanofibers were thoroughly characterized. In detail, scanning electron microscope (SEM) and Fourier transform infrared spectrometer (FTIR) was applied to probe the nanofiber topology and vibrational modes of chemical bonds, respectively. For exploring the potential of PLA/PLX nanofiber membrane in sustained drug delivery for post-surgical site of tumor removal, the tumor killing efficacy of PLA/PLX nanofiber membrane were measured with in vitro MTT assays (for cell proliferation and cell cycle) through their bio-toxicity against model cell line of neuroblastoma, which is the most common cancer among young children.

## Methods

**Fig. 1:**
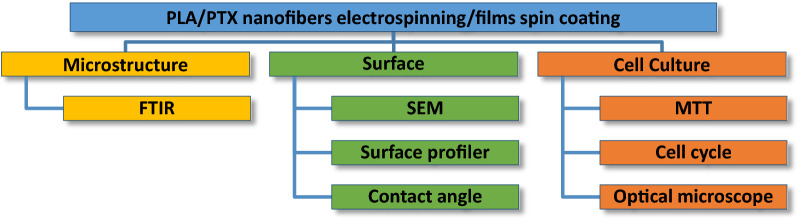
Experiment study for the electrospun paclitaxel mixed PLA nanofibers on top of a thin film of same compositions

The experimental setup for the study PLA/PTX nanofiber and thin films is laid out in Fig. 1. It needs to be noted that either nanofiber or thin films used herein is prepared from a mixture of PLA (40 wt.%) and paclitaxel. In general, nanofiber membrane are fabricated by electrospinning technique while thin film is formed by spin coating on a glass substrate. For material characterizations and biological studies, we place various samples of PLA/PTX nanofiber membrane on top of thin film of same composition which was spin-coated on a glass substrate for convenient measurements in various instruments.

## Substrate and materials

Glass coverslips (Corning 1737) were cut into a dimension of 10 mm × 10 mm by tungsten cutter and then ultrasonically cleaned in KOH (85%), followed by acetone (99.9%), DI water, and finally by alcohol (90%). Each cleaning step took around 10 min. After thorough cleaning, the glass substrates were blown to dry by nitrogen gas.

Poly(l-lactide) (, , Synthesis C_3_H_4_O_2_]_n_, CAS 26161-42-2, FL-94829, Sigma-Aldrich, St. Louis, MO USA) made from lactide ( ) in the form of white to light yellow powder was dissolved in trifluoroacetic acid (TFA, 99%, CF_3_CO_2_H, , CAS 76-05-1, Alfa Aesar, Ward Hill, MA USA) and then continuously stirred for 24 h at room temperature before used for electrospinning. The concentration of PLA solution is listed in Table 1. Paclitaxel (CAS: 33069-62-4, C_47_H_51_NO_14_) purchased from Sigma-Aldrich Inc. (UNI-ONWARD Corp. New Taipei City, Taiwan) in a powder form was dissolved in dimethyl sulfoxide (DMSO, CAS: 67-68-5, C_2_H_6_OS, Sigma-Aldrich, UNI-ONWARD Corp. New Taipei City, Taiwan). To obtain the best quality in the electrospun nanofibers, paclitaxel/DMSO solution (2.5 mg/100 μl (50%) or 5 mg/100 μl (100%)) was mixed with PLA (40 wt.%) solution by using the volumetric ratio of PLA/paclitaxel = 3 ml: 200 μl to form the finalized polymer solution for either spin coating or electrospinning.

**Table 1:** Parameters for deposition of PLA/paclitaxel mixed layer film by spin coating and nanofibers by electrospinning

*Process parameters for PLA/paclitaxel films by spin coating*	
Speed (rpm)	3000
Operational temperature (°C)	Room temperature
Deposition time (min)	4
PLA concentration in TFA (wt.%)	40
Paclitaxel concentration in DMSO	2.5 mg/100 μl (50%), 5 mg/100 μl (100%)
*Process parameters for PLA/paclitaxel nanofibers by electrospinning*	
Voltage (kV)	17
Flow rate (μl/min)	1 × 10^−2^
Syringe outer/inner diameter (mm)	5.0 × 10^−1^/2.6 × 10^−1^
Operational temperature (°C)	Room temperature
Working distance (cm)	14 or 15
Deposition time (s)	> 60
PLA concentration in TFA (wt.%)	40
Paclitaxel concentration in DMSO	2.5 mg/1 ml (50%), 5 mg/1 ml (100%)
Volumetric mixing ratio (PLA: Paclitaxel)	3 ml: 200 μl

### Spin coating

First of all, glass substrates were treated by reactive-ion etching (RIE, 50w, Ar and O_2_ flow rates of 30sccm) for 30 min in order to increase the surface hydrophilicity. Then PLA/PTX thin film with various compositions was produced by spin coating (SWIENCO SP-02, Power Assist Instrument Scientific Corp. Taoyuan, Taiwan) on glass substrate in a solution mixture of PLA and PTX. The optimized spinning speed of 3000 rpm was set to from different types of PLA/paclitaxel thin films. All coating processes were completed within 4 min in order to allow the solution to cover across the entire glass substrate. After coating, we let the sample to dry for around 30 min in atmosphere environment before electrospinning of the polymeric nanofibers on top of the PLA/paclitaxel thin films.

### Electrospinning of nanofiber membrane

The electrospinning of PLA/PTX was carried with commercially available electrospinning system (FES-COE, Falco, Taiwan) which consists of a syringe pump, a power supply and a two-dimensional motorized platform (collector) for the glass substrate. In brief, the solution mixture of PLA and PTX was first drawn into a glass syringe before the eventual deposition onto a substrate. The operational parameters of the electrospinning process are listed in Table 1. It must be noted that the distance between the syringe’s needle tip and substrate is optimized by trial and errors for depositing PLA/PTX nanofiber membrane with uniform thickness. The dimension (diameter) of nano-fibers is controlled by both applied voltage and flow rate. In general, the higher the applied voltage or flow rates, the thinner the fibers were produced. However, exceedingly high voltage often induces arching between the glass substrate and needle, which leads to the breakup of a streaming jet into droplets. Physical parameters in Table 1 were chosen after many rounds of process optimization in order to assure the highly reproducible properties of PLA/paclitaxel nanofibers.

### Characterization

#### Surface morphology of layer film and nanofibers

The diameters of electrospun PLA/paclitaxel fibers were measured from images by scanning electron microscope (SEM, S-3400 N, Hitachi, Japan) with Image J software. For SEM measurement, the voltage of the accelerated electron beam is set at 15 kV and the magnification is chosen to be 2000× for the best resolution. The average and variances of the nanofiber diameters is averaged from 15 to 20 fibers randomly selected across the entire region of any SEM image.

#### Molecular structure

To determine the vibrational modes of molecular bonding in the deposited PLA/paclitaxel layer films and nanofibers, Fourier transformed infrared spectrometer, FTIR (Perkin-Elmer Pentagon 1005, USA) equipped with He–Ne laser as excitation source at 632.8 nm (25 mW)) was used to detect the infrared absorption of the samples in the range of 450–4000 cm^−1^ with a resolution of 1 cm^−1^ in reflection mode. The FTIR spectrum of a plain glass substrate was first probed with FTIR and used as a control for comparing with those of other samples. Moreover, the IR detector is set at the backside of each sample. The spectrum was numerically analyzed with Fityk 0.9.8 software using Gaussian fitting to identify all peaks against the vibrational modes of various types of chemical bonds. Major FTIR absorption peaks of both PLA and paclitaxel from previous studies are listed in Table 2 [4, 6, 14–29].

**Table 2:** Selected FTIR absorption peaks for PLA [14–24] and paclitaxel [14–29]

Wavenumber (cm^−1^)	Mode
*PLA*	
616	COO–bend
806	COO–(out-of-plane)
973	C–N stretching
1022	–CH_2_ (out-of-plane)
1090	C–O stretching
1183	–C–O–C–
1224	–C–O–H–
1305	C–H (out-of-plane)
1339	C–H bending
1434	COO–symmetric stretching
1470	–CH_2_ bending
1601	COO–asymmetric stretching
1746	C=O stretching
2905	C–H stretching
2956	–CH_2_ symmetric stretching
3004	–CH_2_ asymmetric stretching
3568	OH (COOH)
3572	OH (COOH)
*Paclitaxel*	
689	C–H out-of-plane/C–C=O deformation
803–941	C–H in-plane deformation
941–803	C–H in-plane deformation
1049–1090	C–O stretching
1112–1117	O–H in-plane bending in (COOH)
1274	C–N stretching
1330–1380	CH_3_ deformation
1369	CH/NH bend
1444	C=C ring stretching
1579–1652	C–C stretching
1640	N–H bending
1733	(C=O stretching) of amide group
1775	C=O
1777	C=O
2541–2973	CH3/C–H stretching
2860	CH
2909	CH stretch
3066	–CH sp^3^ stretching
3339	N–H/O–H stretching
3568	OH (COOH)
3572	OH (COOH)

#### Contact angle measurement

The contact angles of deposited films were measured from the image of water droplets (1 ml) on the surface of each sample as taken by a CCD camera (Watec, WAT-902B, Watec Corp, Japan). Images were further analyzed by Image J (ver. 1.52k released on 29 January 2019, National Institute of Health US) to obtain the contact angle.

### Cancer cell culture

#### Cell preparation

Human colorectal carcinoma cells (HCT-116) were purchased from Bioresource Collection and Research Center (Hsinchu, Taiwan) for our study. These cells were stored in Ependroff tubes and kept in − 20 °C freezer until use. Before each experiment, a batch of cells was first incubated and shaken in water bath at 37 °C for about 30 min. After temperature equilibration, HCT-116 containing suspension is pipetted into the fresh culture medium (Dulbecco’s Modified Eagle Medium/High Glucose powder, Gibco^®^, Thermo Fisher Scientific, USA) inside a petri dish of 10 cm in diameter. Using the existing established protocol, we cultured HCT-116 in an temperature-controlled incubator (ShelLab, 2424IR, USA) at 37 °C with a supply of 5% CO_2_ and 95% relative humidity. The cells were typically cultured for 2 to 3 days before they were trypsinized and pipetted into clean Ependroff tubes. The entire suspension in Ependroff tubes was then centrifuged for 3 min at 1300 rpm. After centrifugation, the supernatant was discarded and cells containing sediment were resuspended in culture medium before loading into a 24-well plate for subsequent tests. The concentration of seeding cells was set at around 10^5^ cells/500 μl in order to avoid over-crowding or under-growing of cells in the subsequent culture. For cell culture study, all PLA/paclitaxel nanofiber samples were sterilized by UV light for at least 15 min and then placed in a 24-well dish for all biological tests.

#### Cell cycle test

First, PLA/paclitaxel samples were loaded in a 6-well plate and submerged in fresh culture medium before placing inside the CO_2_ incubator. After 2 h incubation, HCT-116 cells at a cell density of around 10^5^ cells/500 μl were loaded into the wells of 12-well plate. Followed by an additional round of incubation for 24 h, the 12-well plates were taken out from incubator and the cell containing suspension were subjected to centrifugation in order to remove supernatant. Next, the cell concentration was reduced from 5 × 10^5^ to 1 × 10^6^ cells/500 μl in every sample. The dilution process involves the addition of Solution 10 lysis buffer (acidic aqueous solution, ChemoMetec) mixed with DAPZ (volumetric ratio 1000:1) and Solution 11 stabilization buffer (basic aqueous solution, ChemoMetec). After the dilution, the stained cells were transferred to A8 slides (NC-Slide A8, ChemoMetec A/S Danmark), which were later analyzed by Chemometec NucleoCounter^®^ (NC-3000, ChemoMetec A/S Danmark) for cell counting and cell cycles assay.

#### Cell viability

MTT is a yellowish and water-soluble tetrazolium salt (3-[4,5-dimethylthiazol-2-yl]-2,5-diphenyltetrazolium-bromide) and is used for measuring viability of cells. In the presence of nicotinamide adenine dinucleotide (generated by active metabolism in cells), the tetrazolium dye contained in MTT is oxidized through the reduction of NADH to NAD^+^, leading to the formation of insoluble formazan with purple color. The insoluble formazan deposited on the cell surface was dissolved with isopropanol or other organic solvents. The dissolved solution has representative optical absorbance peaks at 562 and 650 nm identifiable by a spectrophotometer (Epoch 2, Microplate Spectrophotometer, BioTek Instruments Inc. USA), whose intensities are proportional to the concentration of formazan. As MTT is found in all living cells, its concentration can be used as a reliable marker for quantifying the number of viable cells. The average intensity of both absorption peaks is usually expressed as optical density (OD) which is defined as

1 $$ {\text{OD = log}}_{ 1 0} \left( {\frac{\text{incident light intensity}}{\text{transmitted light intensity}}} \right) \, $$

**Fig. 2:**
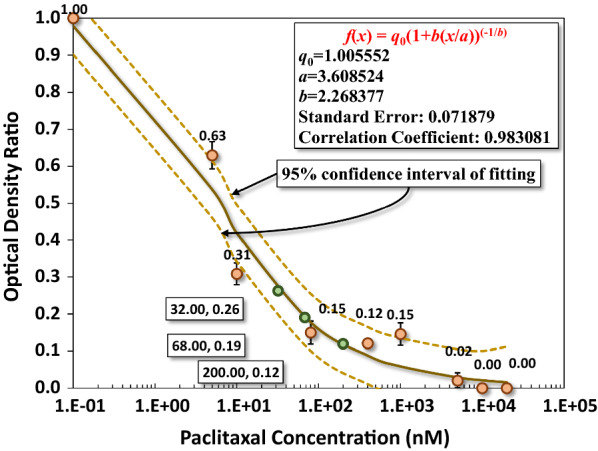
The benchmark test for the optical density ratio from MTT assay on the culture of HCT-116 as a function of paclitaxel concentration in the culture medium

To estimate the amount of released paclitaxel from PLA/paclitaxel mixed nanofiber, we first established a benchmark chart for the optical density from MTT assay on the culture of HCT-116. The culture follows the procedure mentioned earlier. This chart, as shown in Fig. 2, represents the variation of optical density ratios against different concentrations of paclitaxel in the culture media. A regression curve of a rational function with fractional power is numerically determined using CurveExpert Basic 2.0 (Hyams Development, Tennessee, USA). This function provides a good fit to the data with a standard error of 0.0719 and the correlation coefficient 0.9831. The regression provides us with a numerical estimation on the concentration of paclitaxel in the culture medium. For example, the optical density ratio is around 0.12 for the concentration of paclitaxel at 200 nM. This concentration marks the upper bond of a low concentration of paclitaxel for anticancer effects as reported in the literature [38–42]. In Fig. 8, the concentration of paclitaxel released from PLA/paclitaxel nanofibers can be calculated to be 32 and 68 nM for the optical density ratio of 0.26 and 0.19, respectively.

## Results

### Morphology of electrospun PLA

**Fig. 3:**
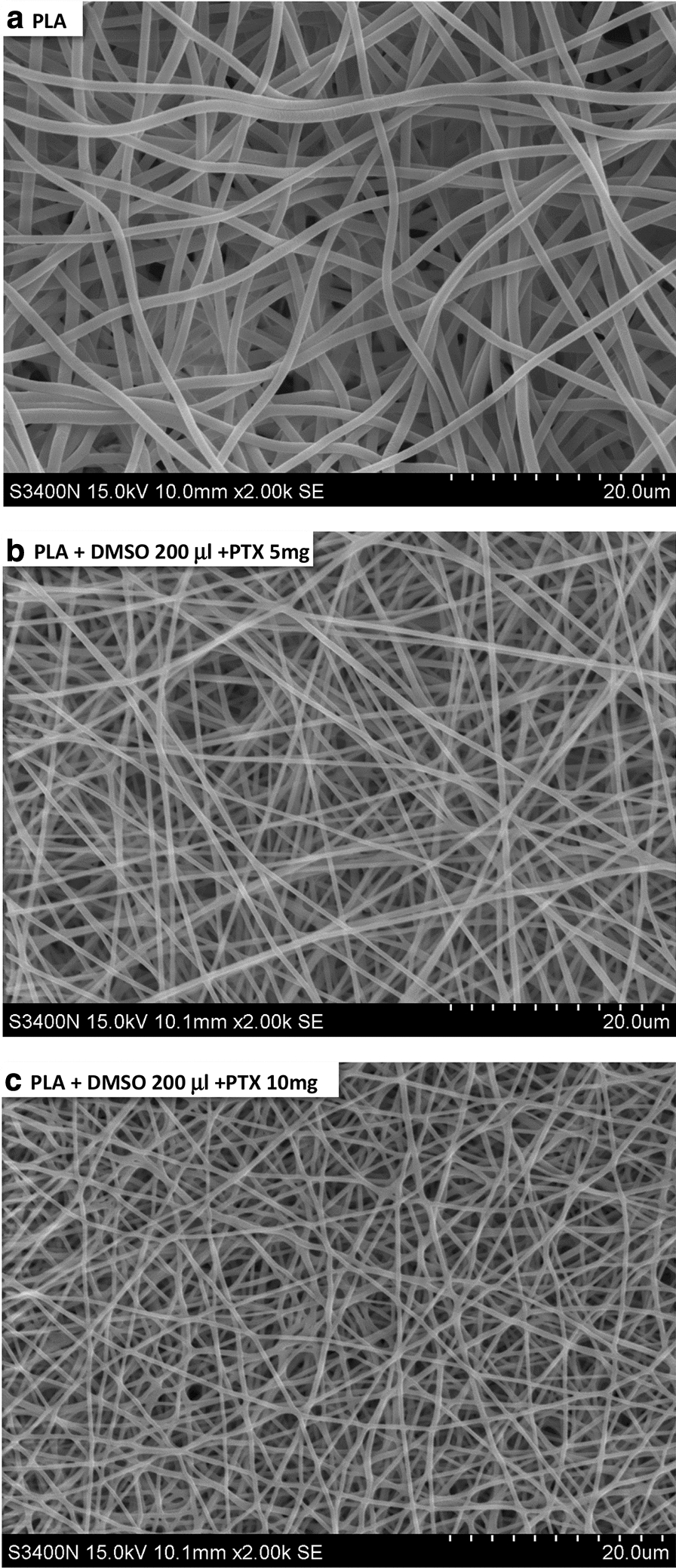
Morphology of electrospun PLA/paclitaxel mixed nanofibers

**Fig. 4:**
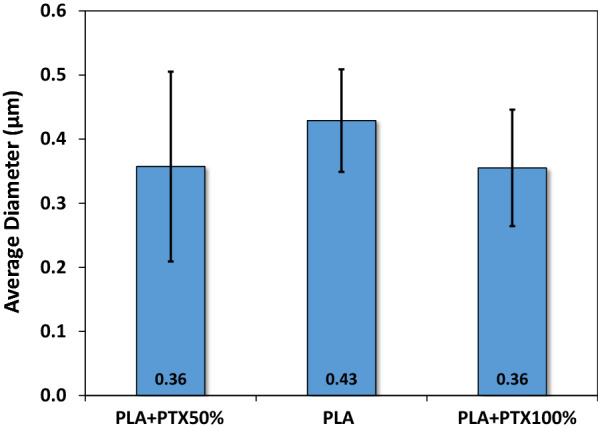
Averaged diameters for electrospun PLA/paclitaxel mixed nanofibers. At least 30 fibers are randomly selected from each image of SEM

SEM images of plain PLA nanofiber and other PLA nanofiber membranes with different concentrations of encapsulated paclitaxel were presented in Fig. 3. The result indicated that nanofibers of pure PLA and PLA incorporated with paclitaxel displayed uniform fiber size and contour length across the entire region of deposition area while the formation of spindles was negligible after the electrospinning process. Moreover, it was shown that plain PLA nanofibers possessed smooth surface morphology without the formation of detectable pores. To quantify the nanofibers’ dimension, the diameter of at least 15 nanofibers were randomly selected from each SEM image of membrane and were measured with image analysis software. The average diameters of nanofibers for three different membrane samples were summarized in Fig. 4. The result indicated that all samples displayed nanofibers with averaged diameters ranging from 0.36 to 0.43 μm.

### Fourier-transform infrared spectroscopy (FTIR)

**Fig. 5:**
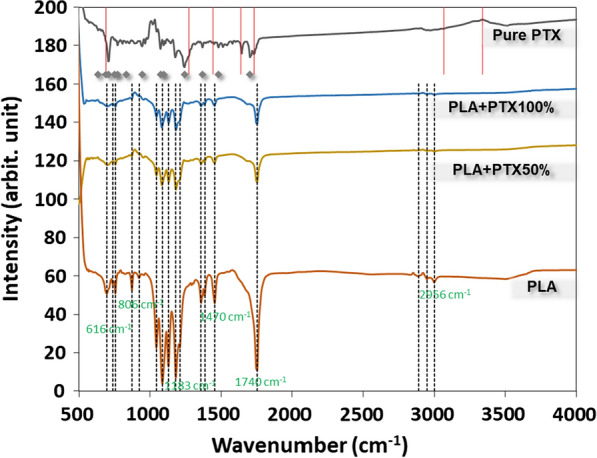
FTIR spectra for electrospun PLA layer films with different concentrations of paclitaxel

FTIR reveals the molecular features on polymeric thin-film through the determination of the unique vibrational modes of various chemical groups. FTIR spectra in the range of 500–3500 cm^−1^ for spin-coated PLA thin films mixed with different concentrations of paclitaxel (PLA+PTX50% and PLA+PTX100%) were shown in Fig. 5, with all absorption peaks fitted by Gaussian fitting in each sample’s spectrum. The FTIR spectra of plain PLA and pure paclitaxel were shown alongside as controls. In general, major FTIR peaks of plain PLA determined by Gaussian fitting included the following vibrational groups: C–O stretch at ~ 1087 cm^−1^, C–O–C at ~ 1183 cm^−1^, O=C–O in ester groups at ~ 1755 cm^−1^, O=C–O stretch in ester groups at ~ 1130 cm^−1^ and CH_3_ at ~ 1458 cm^−1^. The result as mentioned above corresponded well with all the basic chemical groups found along the backbone of PLA. For PTX, major vibrational modes included C-H out-of-plane or C–C =O deformation at 689 cm^−1^, C–H in-plane deformation at 803 ~ 941 cm^−1^, C–O stretching at 1049 ~ 1090 cm^−1^, C–N stretching at 1274 cm^−1^, CH_3_ deformation at 1330 ~ 1380 cm^−1^, C=C ring stretching at 1444 cm^−1^, C–C stretching at 1579 ~ 1652 cm^−1^, N–H bending at 1640 cm^−1^, C=O stretching at 1720 ~ 1727 cm^−1^, (C=O stretching) of amide group at 1733 cm^−1^, CH3/C–H stretching at 2541 ~ 2973 cm^−1^, –CH sp^3^ stretching at 3066 cm^−1^, N–H/O–H stretching at 3339 cm^−1^, agreed with those peaks of pure paclitaxel as reported in the literature [4, 6, 14–29]. Those main and minor vibrational modes of plain PLA and pure paclitaxel were summarized in Table 2.

### Contact angle measurement

**Fig. 6:**
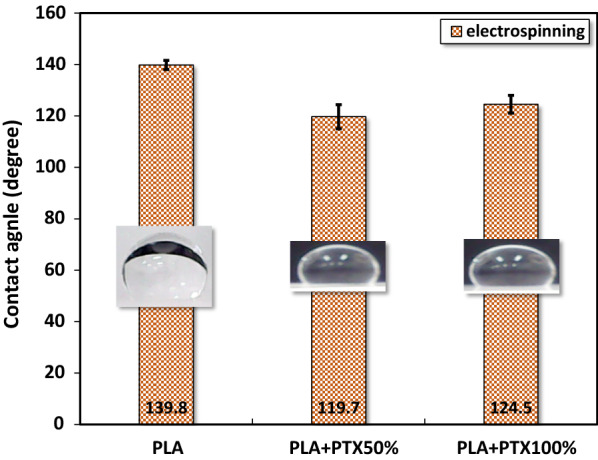
Contact angle for PLA/paclitaxel mixed nanofibers. All photos are images of water drops on nanofibers

The averaged contact angles of PLA, PLA + PTX50% and PLA + PTX100% nanofiber membrane, supported on respective spun coated thin film were shown in Fig. 6. Regardless of the composition, the water contact angles of all samples were larger than 90°, implying high hydrophobicity displayed on these nanofibers. However, the incorporation of PTX into PLA matirx made the nanofiber coated substrate less hydrophobic as show by averaged water contact angles of 119.7° and 124.5° in PLA+PTX50% and PLA+PTX100%, respectively, compared to the higher average of of 139.8° in pure PLA.

### Cell culture

#### Biocompatibility of pure PLA nanofibers and films

**Fig. 7:**
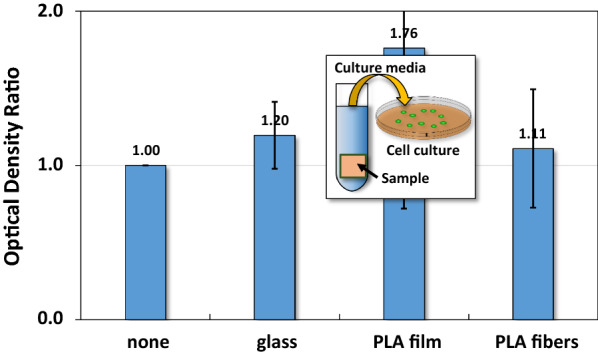
MTT assay for HCT-116 cultured in media from pure PLA films and nanofibers. The optical density is ${ \log }_{10} \left( {\frac{\text{incident light intensity }}{\text{transmitted light intensity}}} \right)$ and the optical density ratio is $\frac{{{\text{OD}}_{\text{specific group}}  }}{{{\text{OD}}_{\text{control}} }}$

To ensure that PLA or PLA/PTX nanofiber membrane impose negligible cytotoxicity, each sample was immersed in plain culture medium for 72 h which was subsequently extracted for culturing HCT-116 cells in vitro. For the ease of direct comparison between the three types of nanofiber membranes, MTT data in terms of optical density ratio ($\frac{{{\text{OD}}_{\text{specific sample}} }}{{{\text{OD}}_{\text{control}} }}$) instead of individual MTT data. Figure 7 showed the optical density ratio of HCT-116 cells determined from MTT assay at 48 h after culturing in the three types of extracted liquid medium. The result indicated that the optical density ratio of HCT-116 cells incubated with extracted culture medium from glass, PLA thin film and PLA nanofiber membrane stays at around one.

#### Toxicity of PLA/paclitaxel mixed nanofibers

**Fig. 8:**
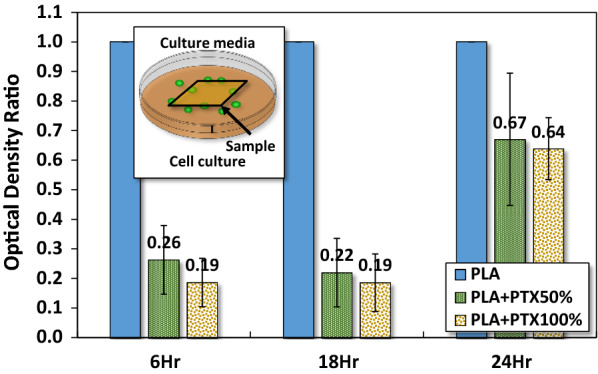
MTT assay for HCT-116 cultured in media with PLA nanofibers mixed with different concentrations of paclitaxel. The optical density is ${ \log }_{10} \left( {\frac{\text{incident light intensity }}{\text{transmitted light intensity}}} \right)$ and the optical density ratio is $\frac{{{\text{OD}}_{\text{specific group}}  }}{{{\text{OD}}_{\text{control}} }}$

Next, PLA or PLA/paclitaxel nanofiber membrane is immersed in liquid medium for the culture of HCT-116. Figure 8 presented the optical density ratio from the MTT assay of HCT-116 cells which were cultured in the presence of PLA or PLA/PTX50% or PLA/PTX100% nanofiber membrane in liquid medium for 6 h, 18 h and 24 h. The control group included HCT-116 cells cultured in liquid medium in the presence of PLA nanofiber membrane. The optical density ratio of HCT-116 cells at each time point was determined as one for the control as mentioned above because there is an absence of paclitaxel in the liquid medium. After 6 h of culture, the optical density ratio of HCT-116 cells was reduced by 74% and 81% in PLA/PTX50% or PLA/PTX100% containing medium, respectively, compared with that for cells in control group (with PLA nanofiber membrane). Similarly, the optical density ratio of HCT-116 cells was reduced by 78% and 81% in PLA/PTX50% or PLA/PTX100% containing medium, respectively, compared with that for cells in control group after 18 h of cell culture. The result indicated that the prolonged release of paclitaxel was maintained by the PLA nanofiber carrier within 18 h of drug adminstration. After 24 h of cell culture, the optical density ratio of HCT-116 cells was reduced by 33% and 36% in PLA/PTX50% or PLA/PTX100% containing medium, respectively, compared with that for cells in control group after 24 h of cell culture.

**Fig. 9:**
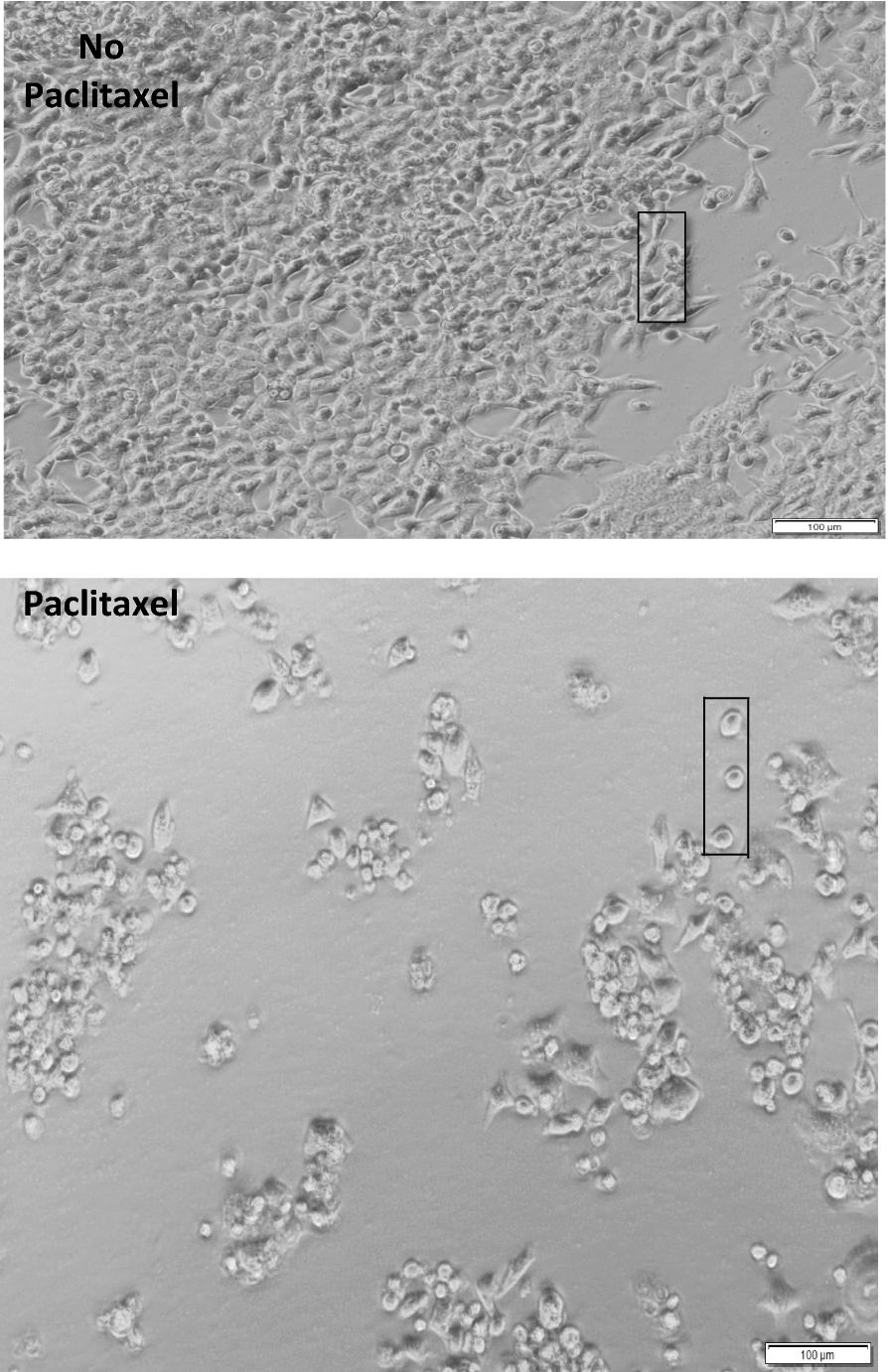
The 100X optical images of cultured HCT-116 on PLA or PLA/PTX50% nanofiber membrane

Figure 9 showed a phase contrast image under 100X magnification of cultured HCT-116 in the presence of PLA or PLA/PTX50% nanofiber membrane in liquid medium after 24 h of cell seeding. In the absence of paclitaxel in PLA nanofiber sample, HCT-116 cells populating at high density on the membrane surface displayed more elongated cell shpe corresponding to the normal morphology of this cell line as shown by the formation of membrane extensions. In contrast, most HCT-116 cells rounded up by transforming into a circular shape rather than the usual stretched and randomly stack up morphology (see the selected view) due to the loss of proliferative activities of the cancerous cells. At the same time, the cell density on the petri dish was significant reduced by PLA/PTX50% compared to that of PLA. The result indicated that paclitaxel acted as an effective toxic molecules presented to HCT-116 cells in the liquid medium before the cells were able to adhere on the nanofiber modified surfaces.

### Cell cycle

**Fig. 10:**
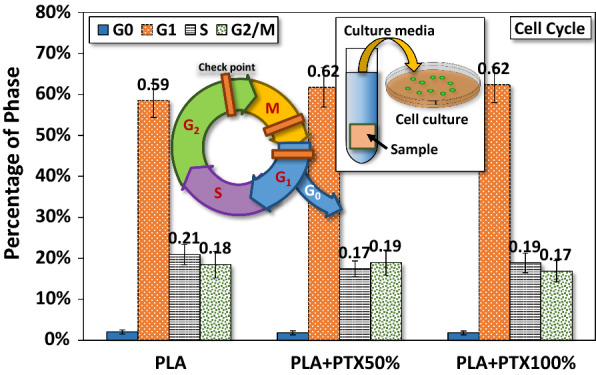
Percentage of different phases in the cell cycle (24 h after seeding) of HCT-116 cultured in media drawn from immersed PLA/paclitaxel mixed nanofibers

Figure 10 showed the percentage of different phases within the cell cycle of HCT-116 cells after 24 h of seeding in the liquid medium pre-incubated with PLA or PLA/PTX50% or PLA/PTX100% nanofiber membrane. In spite of the change in the concentrations of paclitaxel in the nanofiber membrane, HCT-116 in the G_1_ phase (~ 60%) overwhelms all others in other phases of cell cycle. Intuitively, G_1_ phase occurs when cells grow normally through the synthesis of various enzymes and nutrients for getting ready for DNA replication in the S phase. The result indicated that most HCT-116 cells stay in G1 phase 24 h after seeding without going into S phase through the G1 checkpoint. Secondly, around 20% of cells was at either S or G_2_/M phases cultured with liquid medium which was pre-incubated with either PLA or PLA/PTX50% or PLA/PTX100% nanofiber membrane.

Different concentrations of paclitaxel have different impacts on the apoptosis of cancer cells. Under a low concentration (< 200 nM), the cell cycle may not be directly affected as paclitaxel may not be able to alter the overall architecture of the microtubules [10, 31–34]. The cell can still maneuver into prometaphase and arrested at the G2/M phase then followed by p34, cdc2 activation and Bcl-2 phosphorylation, leading to the eventual apoptosis [10, 33–39]. In our cases, the released paclitaxel concentration is estimated at around 32–68 nM for the optical density ratio of 0.26 and 0.19 respectively. It could be concluded that the anticancer effect is attributed to the lower concentration of paclitaxel, which would have a direct impact on the microtubules of HCT-116, e.g., increased difficulty in passing thought the S1 checkpoint but not the overall mitosis [38–42].

## Discussion

The emergence of nanocomposites has led to development of advanced thin-film for drug delivery applications [43]. Moreover, the development of new cancer therapeutics requires complementary molecular carriers with new physiochemical properties [44]. A PLA/PTX nanofiber-based thin film was developed herein for the sustained release of cancer therapeutics. SEM was first applied to probe the structure of PLA based nanofibers. Interestingly, the average diameter of plain PLA nanofibers was larger than that of composite nanofibers including PLA + PTX50% and PLA + PTX100%. The trend as mentioned above is brought about by the lower viscosity and more surface charges of PLA/PTX mixture for electrospinning, leading to overcome the surface tension against the formation of a thinner stretch of plain PLA nanofibers [30]. From the FTIR measurements, PLA + PTX50% and PLA + PTX100% were supposed to display absorption peaks of paclitaxel in addition to those of pure PLA but only the main peaks of PLA were detected. Since the mass percentage of PLA in either PLA + PTX50% or PLA + PTX100% nanofibers is significantly larger than that of paclitaxel, the adsorption perks of paclitaxel with significantly lower signal to noise ratio did not manage to show up in the complexed nanofiber. Numerical fitting, on the other hand, provided information about absorption peaks that belongs to paclitaxel (data not shown). This serves as a confirmation for the presence of paclitaxel in PLA nanofibers.

From the results of contact angle measurements, it was shown that the complex morphology of nanofiber surface and the mesh-like structures of deposited layer likely influence the interaction between the water droplet and underlying substrate. For instance, water eventually sinks into the porous matrix of the pure PLA nanofiber mesh through diffusion and adsorption, leading to the alteration in hydrophobicity compared to pure PLA coated film [45]. Also, the slight reduction of PLA nanofiber’s contact angle upon PTX incorporation is likely caused by the moderation of the solid–liquid interface induced by the complexation between PTX and PLA, as shown in the formation of a hydrogel through the complexation between PLX and amphipathic peptide in aqueous solution [46]. Thus the measurement of water contact angles is only meaningful at the beginning of the test. Nevertheless, the result still can be used as a reference for the relative hydrophilicity among the three types of substrates used herein. The biocompatibility and targeted toxicity of PLA-based nanofiber system were critical to the therapeutic efficacy of the newly developed biomaterial system. Firstly, the MTT results strongly supported that both PLA thin film and PLA nanofiber membrane did not release any toxic debris through its biodegradation, as shown by the unchanged concentration of formazan, relating to cell activity. Interestingly, PLA thin film without the presence of any PLA nanofiber demonstrated the highest cell proliferative activities among all the substrates used herein. The trend as mentioned above was likely caused by the lower hydrophobicity of pure PLA film, in comparison with PLA nanofiber coated substrate, leading to the optimized adsorption of serum protein for promoting cell adhesion and functions [47].

Then MTT assay was used to assess the viability of HCT-116 cells under the influence of PLA/PLX nanofibers. The result strongly supported that both PLA/PTX100% and PLA/PTX50% nanofibers are effective in eradicating cancerous cells. Also, the efficacy of PLA/PTX100% in the eradication of cancerous cells is slightly higher than that of PLA/PTX50% during short term release of paclitaxel. This result implied that the accelerated growth of cancerous cells has led to the elevated depletion of Paclitaxel loading released from the PLA/PTX nanofiber to culture medium. More specifically, the extended proliferation of HCT-116 led to the offset of antineoplastic effect by paclitaxel. However, paclitaxel released from PLA/PTX nanofiber still retained its ability to eradicate HCT-116 cells even after 24 h of cell seeding. In general, paclitaxel has been known to target tubulin, stopping cell divisions through the intervention of microtubule disassembly, mitotic spindle formation and chromosome segregation within cancerous cells. Most importantly, the result of phase contrast microscopy validated the successful eradication of HCT-116 cells in the cell culture medium, leading to the reduced cell population adhered on the PLA/PTX nanofiber immobilized surface. Thus the presence paclitaxel of in the culture medium should have an direct impact on the morphology of cells. Similar nanofiber-based system has been developed from alginate for the controlled release of silver ions in antimicrobial applications [48].

The results from cell cycle assay revealed that a significant proportion of the cultured cells are in the stage of DNA replication and mitosis in G_2_ and M phases, respectively, leading to the separation of replicated DNA and division into two cells. Thirdly, the percentage of cells in the G_0_ phase is smallest (< 2.5%) among all phases of cell cycle. The G_0_ phase is a period that cells put themselves into a quiescent state, neither dividing nor preparing to divide. Cells decide to move into this phase usually due to some adversary factors or peculiar biological situations. Summarizing these results, we can infer that HCT-116 cells were effectively restrained from DNA replication and subsequent cell division under the current level of released paclitaxel from PLA/PLX nanofiber membrane. One important question is how the released paclitaxel can threaten the growth of HCT-116 during in vitro culture? To answer this question, we need a quantitative estimation of the concentration of paclitaxel in culture media, which is empirically estimated by measuring the optical density ratio from MTT assay on the culture HCT-116 in media mixed with various pre-selected concentrations of paclitaxel. Using conational regression analysis, a fitting function can be determined numerically with statistical errors. Once the fitting function is determined, we can estimate the optical density ratio at a specific concentration of paclitaxel.

## Conclusion

In this study, we fabricated a specially designed PLA platform for cell culture. The electrospun PLA nanofibers mixed with different concentrations of paclitaxel in the form of membrane were deposited on PLA spin-coated thin films of the same compositions. The surface morphology of each type of PLA/PTX nanofiber membrane were first probed with SEM, revealing that the average diameter of rather homogeneous PLA or PLA/PTX nanofibers were about the same, under a uniform distribution under the current setup of electrospinning. Moreover, FTIR confirmed the encapsulation of paclitaxel in PLA nanofiber with the presence of both molecular vibrational modes of PLA and paclitaxel components. These PLA/PTX nanofiber membrane is also slightly less hydrophobic, based on the high water contact angle in comparison with that of PLA nanofiber membrane. The effect of the encapsulated paclitaxel on the proliferation and cell cycle of cancerous cells was probed with MTT assay and cell cycle assay for HCT-116 cells in liquid culture media pre-incubated with PLA/paclitaxel nanofiber membrane. The results strongly indicated that paclitaxel has a prolonged impact on the cell proliferation up to 24 h after the seeding. Since the released concentration of paclitaxel is not high (< 100 nM), it has a subtle impact on the cell cycle through the enforcement of G1 checkpoint, with majority of cells in G1 phase at 24 h after the seeding. The results have demonstrated the potential of a new PLA/paclitaxel nanofiber-based system as a sustained release patch for post-surgical drug delivery of cancer therapeutics.

## Data Availability

The data and materials from this study are available from the corresponding authors on reasonable request.

## Abbreviations

PLA: Polylactic acid
PTX: Paclitaxel
FTIR: Fourier-transform infrared spectroscopy
SEM: Scanning electron microscope
